# Assessing Awareness and Knowledge Gaps in Diabetic Distress Among Qatar's Healthcare Providers: A Cross‐Sectional Study

**DOI:** 10.1002/edm2.70117

**Published:** 2025-12-10

**Authors:** Mutwakil Elbidairi, Tatiane Aparecida de Miranda, Camila María Martínez Marte, Ewerton Alves Portela dos Santos, Waseeim Raja, Aboobacher Kandakkeel, Yosria A. I. Ghorab

**Affiliations:** ^1^ Pharmacy Department Hamad Medical Corporation Doha Qatar; ^2^ State University of Western Paraná Cascavel Brazil; ^3^ Pontificia Universidad Católica Madre y Maestra Santiago Dominican Republic; ^4^ Department of Medical Affairs Clinical Studies and Post‐Registration Surveillance (DEAME)/Bio‐Manguinhos‐Fiocruz Rio de Janeiro Brazil; ^5^ Nursing and Inpatient Service Department Hamad Medical Corporation Doha Qatar

**Keywords:** cross‐sectional study, diabetes management, diabetic distress, healthcare professionals, Qatar

## Abstract

**Background:**

Diabetes distress refers to the emotional burden associated with the ongoing management of diabetes. It affects up to 77% of patients with diabetes and is associated with poor glycemic control, reduced adherence to treatment, and lower quality of life. Despite its clinical relevance, diabetes distress (DD) remains under‐recognised by healthcare professionals, particularly in the broader Middle East.

**Objective:**

To assess the awareness and knowledge of diabetic distress among healthcare providers in a tertiary care hospital in Qatar and to identify demographic or professional factors influencing their responses.

**Methods:**

A cross‐sectional survey of 64 healthcare providers at Al Khor Hospital was conducted using an 18‐item questionnaire assessing healthcare providers' Diabetes distress knowledge, consequences, and management familiarity. Associations between knowledge and factors such as specialty, experience, and prior Diabetes distress exposure were analysed using chi‐square or Fisher's exact tests with Bonferroni adjustments for multiple comparisons.

**Results:**

Only 60.3% of respondents had heard of diabetes distress. Significant knowledge gaps existed, particularly among non‐endocrinology professionals and those without prior formal education on diabetes distress. Prior exposure through educational settings was significantly associated with improved knowledge (*p* < 0.05), while clinical experience alone was not.

**Conclusion:**

Substantial knowledge gaps persist in recognizing and managing diabetic distress among Qatar's healthcare providers. Educational exposure was a stronger determinant of knowledge than years of experience. Structured, interdisciplinary training programs are urgently needed.

## Graphical abstract

A survey of 64 healthcare providers in Qatar found that only 60.3% recognized diabetes distress (DD), with notable knowledge gaps among non‐endocrinologists. Past DD education, rather than clinical experience, predicted better awareness (*p* < 0.05). The results underscore the urgent need for interdisciplinary DD training programs to enhance patient outcomes in diabetes care.

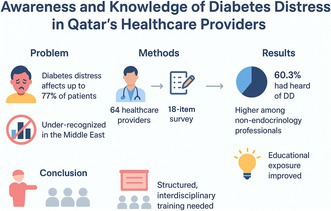

**Abbreviations**
AKH: AL Khor hospital
DD: Diabetes distress
DM: Diabetes Mellitus
HMC: Hamad Medical Corporation
US: United States of America
WHO: World Health Organization

## 1 Introduction

Diabetes distress, also termed “diabetes‐specific distress,” is the emotional burden tied to the rigorous and ongoing management of diabetes. It results from persistent treatment demands and the threat of complications, compounded by social stigma, family strain, and financial burdens [1, 2, 3].

Diabetes distress spans a spectrum of severity and nature, fluctuating over time and often peaking during challenging periods, such as immediately following diagnosis, significant treatment changes, or when complications arise. Heightened general stress can also exacerbate it, potentially progressing to more severe conditions like anxiety or depression if left untreated [4, 5, 6].

Globally, diabetes affects a significant portion of the population, increasing from 422 million adults diagnosed in 2014 [7] to nearly 500 million in 2017. Among them, diabetes distress affects between 42.5% and 77.2% of patients [5]. While some studies report similar prevalence across diabetes types [3], others indicate higher rates among certain subgroups, women with both Type 1 and Type 2 diabetes [5]. A systematic review across 50 studies revealed that about one in four individuals with Type 1 diabetes and one in five with Type 2 diabetes experience high levels of distress, likely impacting their diabetes management [8, 9].

If unmanaged, diabetes distress can lead to adverse physical and mental health outcomes, such as poor self‐management behaviours (e.g., reduced physical activity, unhealthy eating habits, non‐adherence to medications, and infrequent blood glucose monitoring), elevated A1C levels, more frequent episodes of severe hypoglycemia, and a decreased quality of life [10, 11, 12].

Despite its clinical relevance, DD remains under‐recognised in clinical settings, particularly in Qatar and the Middle East [13, 14]. This study aimed to assess the awareness and knowledge of diabetic distress among healthcare providers in a tertiary care hospital in Qatar, and to identify demographic and professional factors influencing their responses.

## 2 Methods

### 2.1 Study Design and Participants

This study is a cross‐sectional survey design targeting healthcare professionals at Al Khor Hospital (AKH), including physicians, diabetes educators, pharmacists, nurses, and dietitians. It used a convenience sampling approach, recruiting physicians, nurses, pharmacists, diabetes educators, and dietitians from a single tertiary hospital (Al Khor Hospital, AKH) in Qatar. This single‐center convenience sampling limits the generalizability of findings to broader healthcare provider populations. Due to the exploratory nature of the study, no a priori sample size calculation was performed. A post hoc power analysis indicated that the achieved sample (*n* = 64) provided > 80% power to detect moderate effect sizes (Cohen's *d* = 0.5) at *α* = 0.05. While adequate for detecting moderate effects, the sample size was insufficient to reliably detect small effect sizes, particularly in multivariable models.

### 2.2 Data Collection Procedure

The questionnaire was adapted from a validated instrument titled “The development and validation of the awareness and knowledge of diabetes distress questionnaire among doctors in Malaysia.” Minor contextual adaptations were made to suit the local healthcare setting (see File S1). Although face and content validity were previously established, no psychometric re‐validation (e.g., Cronbach's alpha) was conducted in the local context. This remains a limitation.

Data were collected through an electronic, self‐administered questionnaire, developed using the SurveyMonkey platform. An invitation email was distributed to eligible staff via their official Hamad Medical Corporation (HMC) email addresses. This email included a brief explanation of the study, a Research Information Sheet, a consent form, and a link to the online survey. Participation was voluntary, and informed consent was implied through the act of accessing and completing the questionnaire.

The questionnaire comprised demographic and professional profile questions, followed by 18 knowledge‐based items divided into three categories: (1) general knowledge about diabetes distress, (2) consequences of untreated diabetes distress, and (3) its management. These items were formulated as affirmative statements, based on a review of relevant literature.

### 2.3 Data Handling and Statistical Analysis

Survey responses were organised and standardised in Microsoft Excel, then analysed using R software, version 4.2.3. Initial analyses included testing for normality using the Shapiro–Wilk test, and missingness was assessed using Little's MCAR test (*p* > 0.05). Visual inspection revealed no systematic patterns. Data were therefore treated as Missing Completely At Random (MCAR), and complete case analysis was applied.

### 2.4 Statistical Analysis

Descriptive statistics included medians and IQRs for continuous variables. Chi‐square or Fisher's exact test was used for categorical associations. When applicable, Mann–Whitney *U* or Kruskal‐Wallis tests were used, and Bonferroni correction was applied for multiple comparisons.

## 3 Results

The study included 64 participants, all of whom were included in the analysis using an available‐case approach. Even though most responses (67.2%) were complete, some missing data were notable, specifically for the variables “How did you hear about Diabetes Distress?” (omitted by six participants) and “Age” (missing in eight cases). Due to the nature of the variables, the relatively low proportion of missing data, and the lack of association among missing patterns, data were assumed to be Missing Completely At Random (MCAR), and no imputation was performed.

All the professionals included in the study were from Al Khor Hospital (AKH). The median age was 44.5 years (IQR 38.0–55.25), and 53.13% (n 34) were male. The professional profile encompasses a range of specialties and qualification levels, with most participants, 40.19% (27), holding a degree in Nutrition, Health, or a related field. Additionally, participants were affiliated with various departments, including pharmacy and clinical pharmacy (25), dietetics or nutrition and dietetics (2), nursing (2), and medical specialties (35), such as nephrology, rheumatology, internal medicine, surgical center, and intensive care unit.

Years of professional experience ranged from 1 to over 10 years; most of whom (57.50%, 56) reported 10 years or more of practice. Regarding patient care, 59.37% (38) saw between 1 and 5 patients with Diabetes Mellitus (DM) per day. Fewer professionals reported seeing over 20 DM patients daily (9.37%, 6) or none at all (3.13%, 2).

Among the professionals surveyed, 76.56% (49) had previously attended a course or workshop on Diabetes, and 60.32% (38) reported having heard specifically about diabetic distress. This prior exposure occurred through various channels, often involving multiple sources. For analytical purposes, these sources were categorised into three settings: *Social* (online website), *work* (managed patients with diabetes distress, someone I know has diabetes distress), *and educational* (medical school lectures, postgraduate lectures, courses/workshops, clinical guidelines, diabetes educators).

**FIGURE 1:**
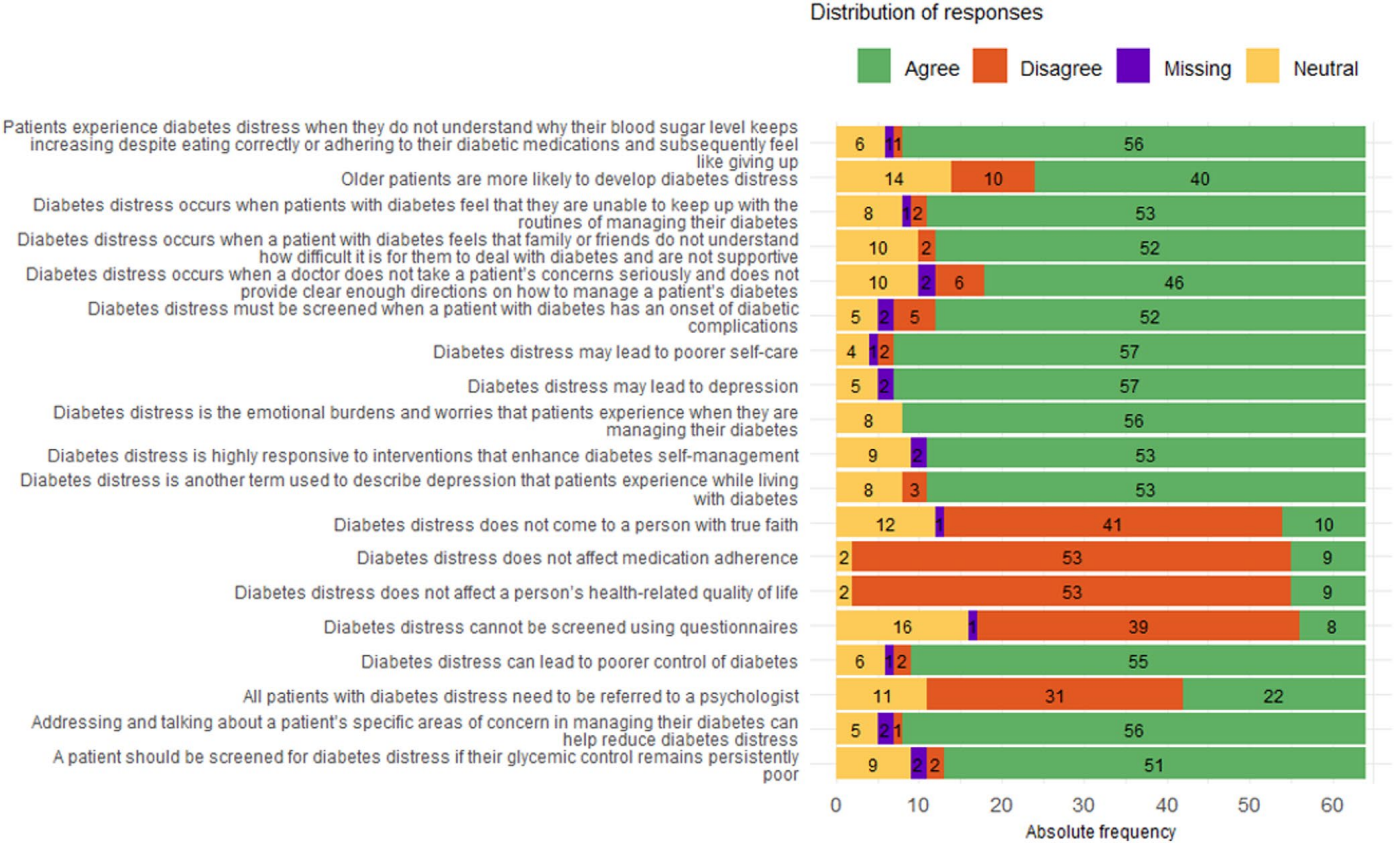
Distribution of responses to diabetes distress knowledge questions (*n* = 64). Data are categorised by response type: True, False, or I Don't Know. *Data source:* Table 1.

Responses to the 18‐item survey are summarised in Figure 1 below. For each question, answers are categorised as “Yes” (believed the statement was true), “No” (believed it was false), and “I don't know” (uncertain or unfamiliar with the statement). Complete proportions are detailed in Table 1 below.

**TABLE 1:** Complete detailed proportions.

No	Questions	True (%)	False (%)	I don't know (%)
*Diabetes distress in general*				
A10	Diabetes distress is the emotional burdens and worries that patients experience when they are managing their diabetes	56 (87.50)	0 (0)	8 (12.50)
A11	Diabetes distress is another term used to describe depression that patients experience while living with diabetes	53 (82.81)	3 (4.69)	8 (12.50)
A12	Older patients are more likely to develop diabetes distress	40 (62.50)	10 (15.62)	14 (21.87)
A13	Diabetes distress occurs when patients with diabetes feel that they are unable to keep up with the routines of managing their diabetes	53 (84.13)	2 (3.17)	8 (12.70)
A14	Diabetes distress occurs when a doctor does not take a patient's concerns seriously and does not provide clear enough directions on how to manage a patient's diabetes	46 (74.19)	6 (9.68)	10 (16.13)
A15	Diabetes distress occurs when a patient with diabetes feels that family or friends do not understand how difficult it is for them to deal with diabetes and are not supportive	52 (81.25)	2 (3.13)	10 (15.62)
A16	Patients experience diabetes distress when they do not understand why their blood sugar level keeps increasing despite eating correctly or adhering to their diabetic medications and subsequently feel like giving up	56 (88.89)	1 (1.59)	6 (9.52)
*Consequences of untreated diabetes distress*				
A17	Diabetes distress can lead to poorer control of diabetes	55 (87.30)	2 (3.17)	6 (9.52)
A18	Diabetes distress does not affect a person's health‐related quality of life	9 (14.06)	53 (82.81)	2 (3.12)
A19	Diabetes distress does not affect medication adherence	9 (14.06)	53 (82.81)	2 (3.12)
A20	Diabetes distress may lead to depression	57 (91.93)	0 (0)	5 (8.06)
A21	Diabetes distress may lead to poorer self‐care (e.g., diet, exercise)	57 (90.48)	2 (3.17)	4 (6.35)
A22	Diabetes distress does not come to a person with true faith (as per my religious believes)	10 (15.87)	41 (65.08)	12 (19.05)
*Diabetes distress management*				
A23	Diabetes distress cannot be screened using questionnaires	8 (12.70)	39 (61.90)	16 (25.40)
A24	A patient should be screened for diabetes distress if their glycemic control remains persistently poor	51 (76.69)	2 (3.22)	9 (14.51)
A25	Diabetes distress must be screened when a patient with diabetes has an onset of diabetic complications	52 (83.87)	5 (9.06)	5 (9.06)
A26	Addressing and talking about a patient's specific areas of concern in managing their diabetes can help reduce diabetes distress	56 (90.32)	1 (1.61)	5 (8.06)
A27	Diabetes distress is highly responsive to interventions that enhance diabetes self‐management	53 (85.48)	0 (0)	9 (14.52)
A28	All patients with diabetes distress need to be referred to a psychologist	22 (34.37)	31 (48.44)	11 (17.19)

The distribution of missing or “Not knowing” responses varied across the questionnaire categories. Overall, the rate of uncertainty was higher in the general and management categories, being approximately 15%, while the consequences of untreated diabetes section showed a rate of 8% (*p* = 0.003, 95% CI, two‐sided). In the management section, 25.4% (16/63) were unsure about the statement “Diabetes distress cannot be screened using questionnaires.” In the general category, 12.5% (8 out of 64) were uncertain about the statement: “Older patients are more likely to develop diabetes distress”, leading to 19.05% (12 out of 63) of the participants to answer “I don't know” to the statement “Diabetes distress does not occur in a person with true faith.”

We also investigated how factors such as specialty, years of experience, and prior awareness of diabetic distress influenced the responses, aiming to guide future continuing education and create an effective learning environment. A summary of the factors impacting each response is shown in Table 2 below.

**TABLE 2:** Univariate analysis of response‐influencing factors.

	No	Age	Gender	Physician	Master^a^	Experience years^b^	Departament^c^	Patients day^d^	Hear about	Educational setting	Work setting	Social setting	Attend a course before
General	A10	0.656*	0.058	1	0.343	0.073	0.097	0.240	0.000	0.000	0.66	0.582	0.377
	A11	0.653	0.076	0.295	0.203	0.134	0.091	0.277	0.009	9.135e‐05	0.264	1	0.712
	A12	0.188	0.345	0.622	0.013	0.047	0.004	0.118	0.069	0.002	0.031	0.099	0.836
	A13	0.588	0.021	1	1	0.115	0.157	0.134	0.006	0.001	0.592	1	0.068
	A14	0.899	0.155	0.834	0.046	0.161	0.015	0.168	0.000	6.828e‐06	0.068	0.069	0.532
	A15	0.735	0.129	1	0.061	0.226	0.060	0.322	0.008	0.000	0.010	0.119	0.438
	A16	0.318	0.025	0.826	0.079	0.058	0.398	0.494	0.006	0.001	0.467	0.522	0.715
Consequences	A17	0.523	0.433	0.433	0.078	0.075	0.292	0.378	0.124	0.041	1	0.266	0.465
	A18	0.191	0.562	0.426	0.003	0.003	0.134	0.536	0.227	0.1807	0.152	0.419	0.238
	A19	0.125	0.637	0.404	0.000	0.003	0.462	0.214	0.227	0.1807	0.608	0.096	0.004
	A20	0.473*	0.355	0.170	0.246	0.226	0.310	0.640	0.062	0.3666	0.573	1	1
	A21	0.668	0.807	0.206	0.191	0.021	0.597	0.658	0.044	0.3215	0.723	1	1
	A22	0.420	0.065	0.301	0.039	0.271	0.001	0.445	0.153	0.027	0.7366	0.837	0.001
Management	A23	0.024	0.935	0.024	0.060	0.185	0.002	0.096	0.095	0.000	0.657	0.006	0.222
	A24	0.266	0.019	1	0.622	0.018	0.114	0.038	0.002	2.452e‐05	0.139	0.061	1.00
	A25	0.414	0.584	0.880	0.271	0.416	0.176	0.445	0.007	0.009	0.537	0.182	0.823
	A26	0.840	0.037	0.825	0.436	0.043	0.499	0.338	0.007	0.049	0.150	1	1
	A27	0.838	0.002	1	0.381	0.097	0.096	0.462	0.070	0.007	0.655	0.266	1
	A28	0.011	0.262	0.053	0.376	0.113	0.000	0.380	0.028	0.001	0.088	0.076	0.729

*Note:* The Wilcoxon rank sum test (Mann–Whitney *U*‐test) was applied to the continuous variables when we want to compare 2 groups. Kruskal‐Wallis was applied to the continuous variables when we want to compare more than 2 groups. Categorical variables are tested by the chi‐squared test, if applicable. Nonetheless, all of them had < 5 observations in at least one category/contingency unit, therefore, analysis was performed using Fisher's Exact test. All tests were two‐sided, IC 95%, alpha = 5.

^a^ Qualification achievement, dichotomized into having a master's degree or not, with a master's degree being considered the “highest qualification.” Hypothesis: More qualified or more experienced professionals have more knowledge about the syndrome under study.

^b^ Years of experience, dichotomized into 1–5 (less experience) and > 5 (more experience). Hypothesis: More qualified or more experienced professionals have more knowledge about the syndrome under study.

^c^ Department, dichotomized into being part of the endocrinology department. Hypothesis: Professionals with specific training have more knowledge about the syndrome under study.

^d^ Patients per day, dichotomized into ≤ 5 and > 5. Hypothesis: More qualified or more experienced professionals have more knowledge about the syndrome under study.

Overall, prior knowledge of diabetic distress was the main factor linked to admitting uncertainty in most questions. This association was especially strong when professionals reported learning about it through formal educational settings. The *general* and *management* sections were the most affected (see Table 2 below).

Having previously attended a course or workshop on Diabetes Mellitus generally did not significantly influence participants' responses. Two exceptions were observed, for the statement “Diabetes distress does not affect medication adherence” (*p* = 0.004), those who had not attended a course were more likely to respond “True” (*p* = 0.044). Similarly, for “Diabetes distress does not occur in a person with true faith (according to my religious beliefs)” (*p* = 0.001), participants without prior training tended to answer “True” (*p* = 0.537) or “I don't know” (*p* = 0.037).

Knowledge gained from work settings generally did not influence response patterns, except for the question “older patients are more likely to develop Diabetes distress” (*p* = 0.031). Work‐related knowledge showed a protective trend against answering “I don't know” (*p* = 0.550), though not statistically significant. Similarly, for “Diabetes distress occurs when a patient with diabetes feels that family or friends do not understand how difficult it is for them to deal with diabetes and are not supportive” (*p* = 0.010), there was a tendency to answer “False” (*p* = 0.07).

Social setting did not significantly impact responses, except for the question “Diabetes distress cannot be screened using questionnaires” (*p* = 0.006), where prior exposure through social contexts showed a non‐significant tendency to answer “True” (*p* = 0.151).

Another factor that showed no significant influence was the total number of patients seen per day. This variable was only associated with the response to the management question “should a patient be screened for diabetes distress if their glycemic control remains persistently poor” (*p* = 0.038). Professionals treating more than five diabetic patients daily were more likely to answer “False” (*p* = 0.981), although this finding was not statistically significant.

Affiliation with an endocrinology department showed some scattered associations, particularly in the *general* section. For the statement “Diabetes distress does not occur in a person with true faith (according to my religious beliefs)” (*p* = 0.001), being part of an endocrinology department was associated with a higher likelihood of responding “False” (*p* = 0.446). Similar trends were observed for “Diabetes distress cannot be screened using questionnaires” (*p* = 0.827) and “All patients with diabetes distress need to be referred to a psychologist” (*p* = 0.013), where endocrinology professionals were also more likely to disagree.

Being from the endocrinology department also appears to reduce the likelihood of answering “I don't know” to certain questions, such as “Diabetes distress occurs when a doctor does not take a patient's concerns seriously and does not provide clear enough instructions on how to manage a patient's Diabetes” (*p* = 0.586), and “older patients are more likely to develop Diabetes distress” (*p* = 0.323). In the latter, there was also a tendency for more “True” responses (*p* = 0.595).

No significant differences were found between physicians and non‐physicians for most responses. The only exception was the statement “All patients with diabetes distress need to be referred to a psychologist” (*p* = 0.053), but post hoc analysis showed no statistical difference between groups (*p* = 1.0).

Educational level showed some associations, particularly in the *consequences of untreated diabetes* section. For the statement “Diabetes distress does not affect a person's health‐related quality of life” (*p* = 0.003), individuals with a higher education level, such as a Master's degree, were more likely to respond “True” (*p* = 0.649) or “I don't know” (*p* = 0.074). A similar pattern was seen for “Diabetes distress does not affect medication adherence” (*p* < 0.001), with more “True” (*p* = 0.114) and “I don't know” (*p* = 0.074) responses. For the statement “Diabetes distress does not occur in a person with true faith” (*p* = 0.039), higher education was non‐significantly associated with the “I don't know” response (*p* = 0.652).

Age and gender had limited influence on participants' responses. Age showed some association with answers in the *management* section, particularly for the statements “Diabetes distress cannot be screened using questionnaires” (*p* = 0.024) and “All patients with diabetes distress need to be referred to a psychologist” (*p* = 0.011).

Gender showed some influence, with female participants less likely to answer “I don't know” to several questions. This was observed in general knowledge items, including “Patients should be screened for diabetes distress if glycemic control remains poor” (*p* = 0.647) and “Diabetes distress results from emotional burdens of managing diabetes” (*p* = 0.546), among others. A similar trend appeared in the management question “Diabetes distress responds well to interventions that support self‐management” (*p* = 0.002).

Responses to the general knowledge covered by questions A10 to A16 were most strongly associated with prior awareness of diabetes distress, particularly when learned through an educational setting, and in some cases through work experience. Other influencing factors included gender, level of education, years of professional experience, and being part of the endocrinology specialty.

Responses to questions on the consequences of untreated diabetes, covered by questions A17 to A22, were influenced by factors such as level of qualification, years of experience, prior knowledge of diabetes distress, especially through educational settings, previous participation in diabetes‐related courses, and affiliation with the endocrinology specialty. However, this category was the least influenced overall by the factors analysed in the professionals' responses.

Understanding the management of diabetes distress, through questions A23 to A28, was mainly influenced by having previously heard about the topic, especially through educational settings. Other influencing factors included the number of diabetes patients seen per day, years of experience, age, gender, being a physician, and working in endocrinology.

In summary, prior knowledge, particularly from the educational environment, greater professional experience, and being part of the endocrinology specialty were the main factors that seem to be associated with better understanding across the sections' general knowledge, consequences of untreated diabetes, and management of diabetic distress syndrome.

## 4 Discussion

This study explored the awareness and knowledge of diabetes distress (DD) among healthcare professionals at a tertiary care hospital in Qatar. Our findings suggest significant variability in understanding across the domains of general knowledge, consequences of untreated DD, and its management. Despite the increasing global emphasis on person‐centered diabetes care, many professionals, particularly those outside endocrinology, demonstrated gaps in recognizing DD as a clinical entity with distinct consequences and treatment pathways. Three findings stand out: (A) nearly 40% of participants had never heard of DD, (B) formal educational exposure rather than years of practice was consistently associated with more accurate responses, and (C) specific misconceptions persist regarding epidemiology and screening, indicating clear targets for educational improvement.

39.68% of respondents had not heard of diabetes distress, and knowledge deficits were evident even among those who had prior exposure. These findings echo international observations that healthcare professionals often conflate diabetes distress with depression or underestimate its impact on clinical outcomes [15]. For instance, a multinational survey revealed that fewer than 50% of primary care physicians regularly screen for DD, often citing time constraints, lack of training, and unfamiliarity with screening tools [16].

Our data also show that educational exposure, particularly through formal teaching or guidelines, was a stronger predictor of accurate responses than clinical experience alone. This highlights a key challenge: despite managing patients with diabetes daily, many providers lack targeted training on the psychological dimensions of care. A recent review emphasized that integrating emotional support into routine diabetes management improves glycemic outcomes, patient adherence, and quality of life [17]. Yet, psychological care remains insufficiently prioritized in medical curricula, particularly in non‐Western contexts [18].

As expected, endocrinologists demonstrated more accurate and confident responses, especially in rejecting misconceptions (e.g., that faith precludes distress, or that all cases require referral to psychologists). This specialisation effect aligns with literature showing that specialists are more likely to adopt patient‐reported outcome measures and apply psychosocial frameworks in diabetes care [11]. Nevertheless, effective DD management requires a multidisciplinary effort, involving dietitians, pharmacists, nurses, and educators, many of whom in our study showed inconsistent knowledge.

Religious and cultural influences may further complicate recognition of DD. A proportion of respondents (19.05%) agreed that distress does not affect people with “true faith,” a belief that may reduce empathy or delay intervention. Prior qualitative studies from Islamic‐majority countries suggest that religious framing can both hinder and facilitate engagement with mental health care, depending on how it is interpreted and integrated into counselling [19, 20, 21]. Future research might explore how religious beliefs act as both barriers and potential buffers to emotional distress, as suggested in studies like Koenig (2012). Importantly, spiritual beliefs may function both as barriers and as buffers; educational content should acknowledge this bidirectionality and equip clinicians to engage with it constructively (Koenig 2012).

Our findings have practical implications. First, continuing professional development (CPD) programs should include structured content on DD and its differentiation from clinical depression. We recommend a brief (≤ 60‐min) module with case vignettes and a five‐item screening practice. Second, routine screening using validated tools such as the PAID (Problem Areas in Diabetes) scale or DDS (Diabetes Distress Scale) should be incorporated into outpatient workflows [20, 22]. Third, culturally contextualised educational materials should address religious beliefs, stigma, and emotional burden, helping professionals navigate complex psychosocial dynamics in diabetes care.

Additionally, emerging evidence supports the use of e‐learning and pharmacist‐led interventions to address knowledge and support gaps in diabetes distress care [23, 24]. Surveys from Western and non‐Western contexts confirm that while many general practitioners and allied health professionals recognize the emotional toll of diabetes, they still underutilize tools and referrals due to low confidence or system‐level constraints [25].

## 5 Strengths and Limitations

A major strength of this study is its focus on an understudied region, adding local data to the global discussion on diabetes distress. Including multiple professions offers a broader view of knowledge gaps across healthcare teams. However, the study has some limitations. The single‐center design might limit how well the results apply to other settings, and the self‐reported questionnaire could be affected by response or recall bias. Also, we did not use a validated screening tool (e.g., PAID or DDS‐17), which might have limited the depth of clinical insight. Furthermore, the findings were not triangulated with clinical outcomes or patient perspectives. Although gender appeared to influence confidence in knowledge, this aspect was not examined in depth and warrants further exploration. This study relied only on providers' self‐reports without cross‐checking with patient outcomes (e.g., HbA1c changes after DD intervention) or qualitative patient feedback. Future research should incorporate mixed methods approaches to evaluate the real‐world impact.

## 6 Conclusion

In conclusion, although healthcare professionals in Qatar are aware of diabetes distress, significant knowledge gaps remain, especially in management strategies and belief‐based misconceptions. Structured, interdisciplinary training and culturally sensitive education are crucial to provide professionals with the tools to address this often‐overlooked aspect of diabetes care. Future studies should assess how targeted educational programs influence both provider practices and patient outcomes. We recommend integrating DD screening tools such as PAID or DDS into routine diabetes care workflows, mandatory CME training on psychosocial diabetes care, and culturally adapted educational programs specifically designed for healthcare professionals in the region.

## Author Contributions

M.E.: Conceptualization, data collection, writing – original draft. T.A.M.: Data analysis, writing – review and editing. C.M.M.M.: Data curation, writing – review and editing. E.A.P.S.: Methodology, supervision. W.R.: Survey coordination, literature review. A.K.: Data management, validation. Y.A.I.G.: Institutional coordination, critical review. All authors have read and approved the final manuscript.

## Ethics Statement

This study was approved by the Medication Safety and Quality Center under the number MRC‐01‐23‐209. Informed consent was implied through voluntary participation in the anonymous survey. All procedures were conducted following the Declaration of Helsinki.

## Conflicts of Interest

The authors declare no conflicts of interest.

## Supporting information


**File S1:** Questionnaire. The 18‐item survey instrument was used to assess healthcare providers' knowledge of diabetic distress.

## Data Availability

The data that support the findings of this study are available from the corresponding author upon reasonable request.
