# Supplementary material for: Assessing Awareness and Knowledge Gaps in Diabetic Distress Among Qatar's Healthcare Providers: A Cross‐Sectional Study
Source: Endocrinol Diabetes Metab. 2025 Dec 10;9(1):e70117. doi: 10.1002/edm2.70117 (PMC12690494; doi:10.1002/edm2.70117)
Supplement: Supplementary file 1 — File S1: Questionnaire. The 18‐item survey instrument was used to assess healthcare providers' knowledge of diabetic distress. [file EDM2-9-e70117-s001.docx]

**Diabetes distress questionnaire**

This study aims to assess healthcare providers' awareness and knowledge gap about diabetic distress, to incorporate the diagnosis of Diabetes Distress into their routine practice when examining diabetic patients.

A1. Age _ year-old

A2. Gender

- Male
- Female

A3. Qualification achieved

- Bachelor’s in medicine and surgery
- Master
- Bachelor’s in nutrition, health, or a related field
- Others (please specify)

A4. how many years of experience (post-full registration)?

- < 1 year
- 1 – 5 years
- 6 – 10 years
- 10 years or above

A5. What is your current department /unit?

- Dietitian
- Medicine
- Staff clinic
- Endocrinology

A6. On average, how many patients with diabetes mellitus do you see in a day?

------- patients/day

A7. Have you attended a course or workshop about diabetes mellitus before?

- Yes
- No

A8. Have you heard of diabetic distress before?

- Yes
- No (if not please skip A9 and proceed to the next one)

A9. How did you hear about diabetes distress? (You may choose more than one answer)

- I have managed patients with diabetes distress before
- A patient who knew about diabetes distress told me
- A family member told me
- Someone I know has diabetes distress
- From medical school lectures
- From postgraduate lectures
- By attending courses/workshops about diabetes
- From clinical practice recommendations/ guidelines
- Online website
- Others (please specify) -------

Please tick ( )one response for each statement:

| No | Questions | True | False | I don’t know |
| --- | --- | --- | --- | --- |
| **Diabetes distress in general** | | | | |
| A10. | Diabetes distress is the emotional burdens and worries that patients experience when they are managing their diabetes |  |  |  |
| A11. | Diabetes distress is another term used to describe depression that patients experience while living with diabetes. |  |  |  |
| A12. | Older patients are more likely to develop diabetes distress. |  |  |  |
| A13. | Diabetes distress occurs when patients with diabetes feel that they are unable to keep up with the routines of managing their diabetes. |  |  |  |
| A14. | Diabetes distress occurs when a doctor does not take a patient’s concerns seriously and does not provide clear enough directions on how to manage a patient’s diabetes |  |  |  |
| A15. | Diabetes distress occurs when a patient with diabetes feels that family or friends do not understand how difficult it is for them to deal with diabetes and are not supportive |  |  |  |
| A16. | Patients experience diabetes distress when they do not understand why their blood sugar level keeps increasing despite eating correctly or adhering to their diabetic medications and subsequently feel like giving up |  |  |  |
| **Consequences of untreated diabetes distress** | | | | |
| A17. | Diabetes distress can lead to poorer control of diabetes. |  |  |  |
| A18. | Diabetes distress does not affect a person’s health-related quality of life |  |  |  |
| A19. | Diabetes distress does not affect medication adherence. |  |  |  |
| A20. | Diabetes distress may lead to depression. |  |  |  |
| A21. | Diabetes distress may lead to poorer self-care ( e.g: diet, exercise ) |  |  |  |
| A22. | Diabetes distress does not come to a person with true faith ( as per my religious believes ) |  |  |  |
| **Diabetes distress management** | | | | |
| A23. | Diabetes distress cannot be screened using questionnaires. |  |  |  |
| A24. | A patient should be screened for diabetes distress if their glycemic control remains persistently poor. |  |  |  |
| A25. | Diabetes distress must be screened when a patient with diabetes has an onset of diabetic complications. |  |  |  |
| A26. | Addressing and talking about a patient’s specific areas of concern in managing their diabetes can help reduce diabetes distress. |  |  |  |
| A27. | Diabetes distress is highly responsive to interventions that enhance diabetes self-management. |  |  |  |
| A28 | All patients with diabetes distress need to be referred to a psychologist |  |  |  |
